# Supplementary material for: Tead1a Initiates Transcriptional Priming Through the TEAD1a/YAP‐Notch1‐Spi1/Cebpα Axis to Promote Neutrophil Fate
Source: Adv Sci (Weinh). 2025 Aug 28;12(41):e05441. doi: 10.1002/advs.202505441 (PMC12591127; doi:10.1002/advs.202505441)
Supplement: Supplementary file 4 — Supplementary Dataset [file ADVS-12-e05441-s004.zip › 3-2.zip!/3-2/02-2.cluster/cluster3/Enrich/ego_CC.pdf]

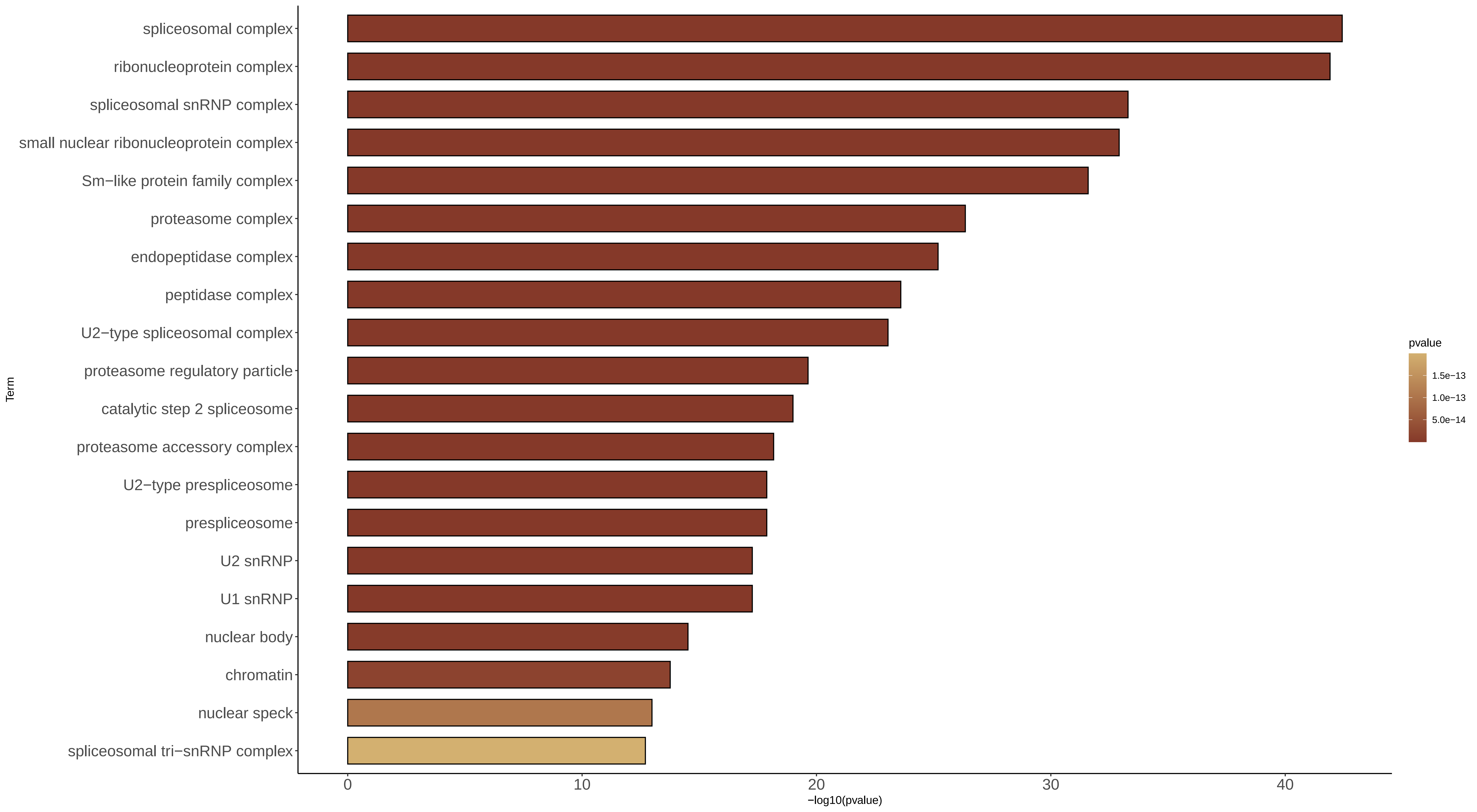

spliceosomal complex
ribonucleoprotein complex
spliceosomal snRNP complex
small nuclear ribonucleoprotein complex
Sm−like protein family complex
proteasome complex
endopeptidase complex
peptidase complex
U2−type spliceosomal complex
proteasome regulatory particle
catalytic step 2 spliceosome
proteasome accessory complex
U2−type prespliceosome
prespliceosome
U2 snRNP
U1 snRNP
nuclear body
chromatin
nuclear speck
spliceosomal tri−snRNP complex
Term
0
10
20
30
40
−log10(pvalue)
pvalue
1.5e−13
1.0e−13
5.0e−14
